# Perspective—Electrochemical Sensors for Neurotransmitters and Psychiatrics: Steps toward Physiological Mental Health Monitoring

**DOI:** 10.1149/1945-7111/ac5e42

**Published:** 2022-04-19

**Authors:** Marjon Zamani, Tatum Wilhelm, Ariel L. Furst

**Affiliations:** *Department of Chemical Engineering, Massachusetts Institute of Technology, Cambridge, Massachusetts*—*02139, United States of America*

## Abstract

Therapeutic monitoring of neurotransmitters (NTs) and psychiatric medications is essential for the diagnosis and treatment of mental illness. However, in-vivo monitoring of NTs in humans as well as continuous physiological monitoring of psychiatrics have yet to be realized. In pursuit of this goal, there has been a plethora of work to develop electrochemical sensors for both in-vivo NT monitoring as well as in-vitro detection of psychiatric medications. We review these sensors here while discussing next steps needed to achieve concurrent, continuous physiological monitoring of NTs and psychiatric medications as part of a closed-loop feedback system that guides medication administration.

In the United States, there are over 50 million adults diagnosed with mental illnesses^1^ This number extends to 2 billion people globally who suffer from mental illness, which is estimated to impose an economic burden of $16 trillion by 2030.^2^ A wide range of medications exist to treat mental illness, including antidepressants, antipsychotics, anti-anxiety medications, and mood stabilizers. The one common factor among mental illness medications is that they all target receptors for neurotransmitters (NT) that modulate the intercellular levels of NTs. NTs are endogenous chemical messengers used for communication between neurons. Abnormal concentrations and general dysfunction of NTs in the central nervous system has been linked to multiple psychiatric illnesses.^3^ Targeting these NTs is crucial in the treatment of these illnesses, but their monitoring in humans has yet to be realized. This ability is crucial because different psychiatric medications have different mechanisms of action (MOA); therefore, proper treatment is contingent on a drug’s MOA being appropriate for an individual’s unique NT imbalance. Currently, no clinically approved molecular diagnostics to ascertain an individual’s NT signature exist. Another key component of treatment for some mental health patients is drug monitoring. This is clinically indicated to optimize dosing for individuals who take drugs that have a narrow therapeutic range, such as lithium.^4^ Drug monitoring is also being explored as a method to combat medication nonadherence, which is common for many individuals struggling with severe mental health illnesses. For example, in the case of schizophrenia, nonadherence to medication accounts for 40% of all rehospitalization costs, and only 1 in 3 patients fully adheres to their medication regimen.^5^

While there exist numerous reviews for electrochemical NT monitoring,^6–8^ this is the first comprehensive review of both NT as well as psychiatric drug monitoring. Ultimately, the goal of those developing sensors for NT and psychiatric drug monitoring is that diagnosis and treatment of psychiatric illness will consist of continuous, physiological monitoring of neurotransmitters and administered psychiatric medications (Fig. 1). The results of this monitoring would be placed in a closed-feedback loop that would enable informed treatment administration. While such a device has yet to be realized, there is a plethora of work that has been done to achieve in-vivo detection of neurotransmitters in animal models, as well as in-vitro detection of psychiatric medications. The majority of these sensors are electrochemical due to the to the precision, sensitivity and portability of electrochemical sensors. Unlike other reviews, we provide the unique perspective of the development of these sensors and the future work that is needed to enable deployment of these technologies in an integrated, closed-feedback control system in humans. This includes a review of wearable technologies that have been developed in pursuit of this goal. Furthermore, this is the only review that describes the detection of all classes of psychiatric medications, rather than focusing on a particular class (i.e. antipsychotics).

## In-Vivo Detection of Neurotransmitters

Electrochemistry is an ideal tool for monitoring NTs because of its portability, sensitivity and ease-of-use.^8^ This transduction method is sufficiently sensitive to detect target concentrations of NTs in the brain (nanomolar).^8^ Electroactive NTs, such as dopamine (DA) and serotonin (5-HT), can be directly detected electrochemically. A number of voltammetry techniques have been used for direct NT detection, most commonly using the techniques fast scan cyclic voltammetry (FSCV) or differential pulse voltammetry (DPV). These methods offer the milli-second timescale resolution needed to capture transient NT activity.^8–11^ A major challenge of direct NT detection is the interference of other species such as uric acid (UA) and ascorbic acid (AA).^12^ Distinguishing between NTs is also a concern, as DA, 5-HT and norepinephrine exhibit similar oxidation potentials while residing in the same brain regions.^10^ One study by Zhu et al. successfully achieved simultaneous detection of DA and 5-HT in-vivo in a mouse brain.^13^ Oxidation peaks of the two molecules were distinguished using DPV. These sensors achieved detection limits of 50 nM for DA and 20 nM for 5-HT, with linear ranges of 0.05–60 *μ*M for DA and 0.1–100 *μ*M for 5-HT. The sensor exhibited selectivity against AA and homovanillic acid due to the ability of DPV to differentiate between the oxidation of DA, 5-HT, AA and homovanillic acid. Detection was achieved using a graphene-iron-tetrasulfophthalocyanine (GR-FeTSPc)-coated carbon fiber microelectrode (GR-FeTSPc/CFME). Carbon-based electrodes are often used due to their electrical conductivity, stability, and low cost.^14,15^ Incorporation of nanomaterials is advantageous, as these materials have an increased surface area compared to planar electrodes. Further, they allow for fast charge transfer and electro-catalytic behavior for detection of NTs.^9^ Despite its advantages, native carbon fiber is heterogeneous; surface modifications can improve electrode homogeneity and sensitivity.^16^ The use of microelectrodes is crucial to minimize the inflammatory response caused by electrode insertion, a natural defense mechanism to a foreign body.^9^ The inflammatory response is one of the biggest issues with in-vivo NT sensing, as it can interfere with sensor performance and cause surrounding tissue damage. To mitigate the effects of this response, the development of biocompatible, conductive micro- and nano- materials remains an important goal.

**Figure 2.**
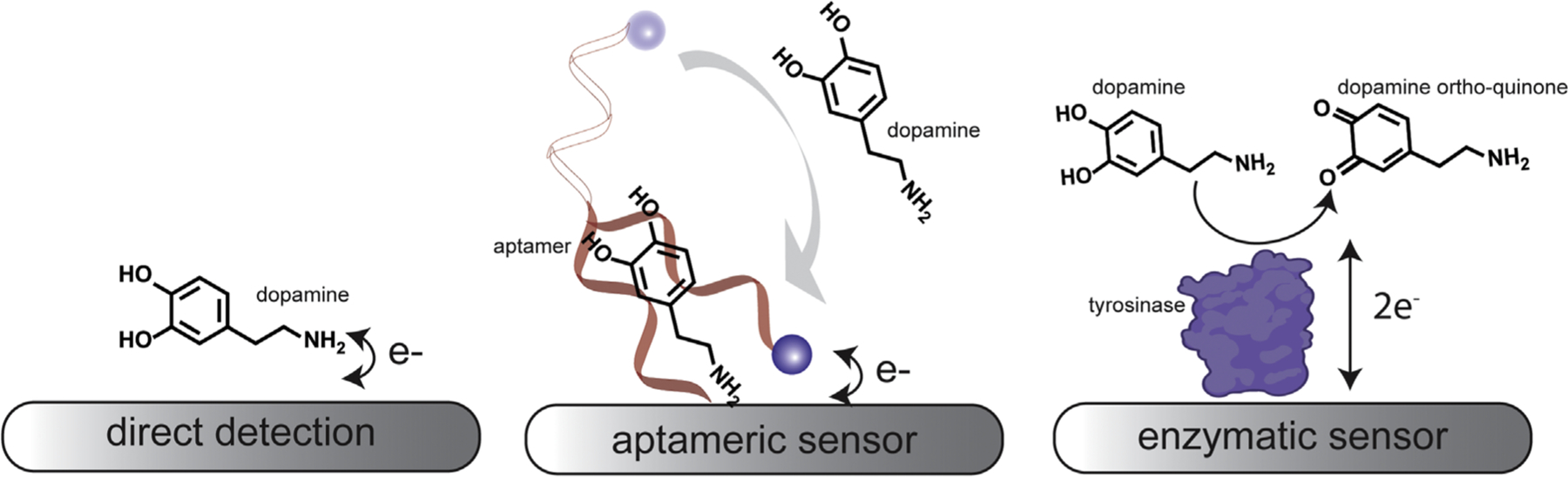
Schematic of electrochemical sensors for neurotransmitter detection.

Many NTs, including glutamate (Glu), GABA, norepinephrine (NE) and acetylcholine (ACh) are not electroactive, precluding their direct electrochemical detection and necessitating the application of specific recognition elements (i.e. biorecognition elements) for detection (Fig. 2).^10^ The two largest classes of indirect NT detection are electrochemical enzymatic biosensors, and aptameric biosensors (E-AB). Both of these sensors can generally be categorized as electrochemical biosensors, which are advantageous because they allow for biological-level specificity, which is difficult to achieve with non-biological recognition elements.^17^

Enzymatic biosensors generally involve immobilized enzymes at the electrode surface. These enzymes generate an electrochemical signal in response to an analyte.^10^ Enzymes can be chosen that are selective for the desired analyte, making them desirable for electro-chemical detection of non-electroactive NTs.^10^ These sensors can also quickly and precisely measure low analyte concentrations in tissue extracellularly in almost real time.^18^ To detect gluamate, for example, the enzyme glutamate oxidase oxidizes glutamate and generates hydrogen peroxide (H_2_O_2_). Although glutamate is non-electroactive, H_2_O_2_ is readily detected electrochemically, enabling quantification through amperometry to measure H_2_O_2_ concentration.^10^ In another study, immobilized glutamate oxidase on an electrode coated with a chitosan polymer matrix enabled glutamate detection with a limit of detection (LOD) of 44 nM and linear range of 5–150 *μ*M.^19^ The polymer matrix served as a selectively permeable membrane that minimized interference from ascorbic acid (AA). This sensor enabled in-vivo detection of glutamate in rat brains and was stable for 4 h. Though 4 h stability represents an important first step for in-vivo deployment, significantly longer times will be necessary for applications in humans. Limited stability of enzymatic biosensors due to enzyme denaturation is a common challenge with using enzymatic biosensors for in-vivo detection. To combat the degradation of the enzyme coating, Doughty et al., achieved NT recordings over 16 weeks in rodent brains by developing a replaceable microwire biosensor where a fresh biosensor was placed in the cannula for each in-vivo measurement.^20^ They used these biosensors to detect GABA by immobilizing GABAse and glucoase oxidase (GOx). The GOx produced *α*-keto to faciltiate the conversion of GABA to hydrogen peroxide by GABAse, which was detected electrochemically. Using this platform, GABA was successfully monitored over a concentration range of 0–500 *μ*M. They improved sensor selectivity by coating the biosensor with a semipermeable membrane (M-phenylenediamine) and by utilizing spatially-multiplexed probes to subtract out background signals from basal catalytic activity. Additional enzymes are used to detect other NTs such as DA and norepeinephrine (NE). Tyrosinase catalyzes dopamine to o-dopaquinone and NE to NE quinone, which are electrochemically detected. One study used tyrosinase to detect NE with an LOD of 196 nM and a linear range of 1–200 *μ*M and distinguished that molecule that from dopamine via DPV in synthetic urine.^21^ Another example involved tyrosinase immobilization in a biopolymer and chitosan matrix.^22^ This platform was used to detect dopamine with a 1 nM LOD and a linear range of 10 nm–220 *μ*M in live rats. The selectively-permeable immobilization matrix allowed for selectivity against AA, UA, 5-HT, NE and L-DOPA. Laccase, which catalyzes oxidation of phenols to quinones, has also been used to detect dopamine levels in synthetic urine. Laccase was immobilized on nanostructured MoS_2_ electrodes to yield a sensor with an LOD of 0.67 *μ*M and a linear range of 1.33–13.32 *μ*M.^23^ Finally, laccase, which catalyzes serotonin oxidation, was used to detect serotonin with an LOD of 48 nmol with a linear range of 0.1–200 *μ*M.^24^

While not as widely used as enzymatic biosensors, aptameric sensors have also been applied to detect NTs. Apatamers are short (20–100 bp) nucleic acids that specifically bind to a compound, resulting in a conformational change of the aptamer.^25,26^ The majority of E-ABs are RNA-based due to their superior ability to undergo a conformational change upon target binding.^25^ The majority of E-ABs are used to detect dopamine. Even though the direct electrochemical detection of dopamine is possible, E-ABs are used to improve sensor selectivity, which is necessary because dopamine oxidizes at similar potentials to 5-HT and NE, which are other NTs that reside in the same brain regions as dopamine.^10^ Two classes of aptamer-based sensors have been developed for dopamine detection. One class enables direct oxidation of dopamine by capturing the molecule adjacent to the electrode surface. In one example of such an E-AB, an RNA aptamer was immobilized on gold microwires for the detection of dopamine. The linear range of detection was 100 nM–5 *μ*M in the presence of competitors such as AA and UA; however, this sensor was only tested in-vitro.^27^ The selectivity of the sensor was found to be improved by applying a Nafion coating to the electrode following aptamer immobilization. Another class of aptameric sensors employed a redox-labeled aptamer to enable indirect dopamine detection.^25^ A redox-labeled aptamer is immobilized on a gold electrode surface; binding of the aptamer to dopamine results in a conformational change that brings the redox label closer to the electrode, resulting in an increase in signal from the redox tag.^26^ One study applying this technique used a redox-labeled aptamer to detect DA with an LOD of 60 pM and a linear range of 0.1 nM–10 nM in-vitro.^28^ Redox-labeled aptamers are also necessary for the detection of non-electroactive NTs such as glutamate, GABA, serotonin and norepinephrine. A DNA-based aptasensor was used to detect glutamate with a limit of detection of 1.3 fM and a linear range between 10 fM and 1 nM in artificial cerebral spinal fluid (CSF) and human serum.^29^ As a final example, a DNA aptasensor was used to detect serotonin with a limit of detection of 0.017 fM and a linear range of 1 pM–10 nM in rat CSF.^30^ There have yet to be aptamers developed to recognize GABA and NE. Yet, E-ABs have been shown to have limited stability in-vivo due to aptamer degradation as well as sensor drift due to electrode fouling, necessitating drift-correction.^9,26,31^

While the efficacy of many NT sensors has not been demonstrated in humans, monitoring of adenosine, a neurochemical, has been achieved in the human brain.^32^ The ability to detect this molecule in the human thalamus demonstrates that in-vivo molecular sensing in the human brain can be performed electrochemically. The researchers used boron-doped diamond electrodes that had comparable sensitivity to carbon electrodes but improved longevity as compared to prevalent platforms. These electrodes were tested in patients undergoing surgery for tremor disorders, where oxidation peaks consistent with adenosine at 1.5 V and 1.0 V were observed following stimulation. This important study demonstrated the first electrode capable of sensing evoked changes in the extracellular levels of neurochemicals in the human brain, demonstrating a critical proof-of-concept for in-vivo NT detection in humans.

## Electrochemical Detection of Psychiatric Medications

During the treatment of mental illness, medication non-adherence and a narrow therapeutic range of some psychiatric medications contribute to treatment failures. Therefore, in addition to detection of NTs, monitoring of psychiatric medications is important for medication management. Such monitoring ensures proper medication adherence and drug therapeutic range. However, neurological drugs, including psychiatrics, can be difficult to analyze, as they often require detection of several analytes simultaneously in complex sample matrices with high precision.^31^ The electrochemical detection of psychiatric medications presents a promising alternative to traditional analysis methods, as electrochemistry offers highly sensitive and rapid ways to analyze complex samples.^33^

Psychiatric drugs can be divided into several different categories based on the type of illness they treat. These include antidepressants, anti-anxiety medications, stimulants, antipsychotics, and mood stabilizers. These medications, often called psychotropic drugs, affect the way in which receptors, transporters, and enzymes function in the body.^34^ Electrochemical sensors have been developed for^35^ antipsychotics,^36–42^ anti-anxiety medications,^35^ antidepressants^43–50^ and the mood stabilizer lithium.^51^ However, electrochemical detection can be difficult because without sample preparation, there are often interfering signals.

Antipsychotics are often used to alleviate symptoms of psychosis in schizophrenia or mania in bi-polar disorder; it has been demonstrated that therapeutic monitoring of atypical antipsychotics is useful to maximize treatment efficacy while minimizing unwanted side effects.^52^ There are a number of different antipsychotics; their electroactive nature enables their detection, which has been extensively reviewed.^42^ In a recent example, a glassy carbon electrode modified with tannic acid and carbon nanotubes was used to detect the common antipsychotic clozapine. Their sensor had a limit of detection of 0.55 nM, a linear range of 55 nM–375 nM and was functional in serum and urine samples.^38^

Anti-anxiety medications alleviate symptoms of anxiety in mental health disorders. A recent example demonstrated electro-chemical detection of the anti-anxiety medication clonazepam.^35^ The group deposited gold nanoparticles on an electrode in such a way to produce a film of gold nanostructures. These nanostructures enabled direct detection of clonazepam with a limit of detection of 550 nM and a linear range of 1–9 *μ*M and was functional in artificial blood.

Antidepressants alleviate symptoms of depression. A recent example demonstrated electrochemical detection of the antidepressant imipramine.^46^ A metal chalcogenide-carbon composite electrode was used for the direct detection of imipramine with a LOD of 4 nm and a linear range of 0.01–51.8 *μ*M. This sensor was suitable and selective against interferents in blood serum as well as urine samples.

Lithium is a mood stabilizer commonly used to treat bipolar disorder. Therapeutic monitoring of lithium is essential due to its narrow therapeutic range. Its electrochemical detection has been extensively reviewed.^51^ While a number of lithium sensors have been used in urine, blood and serum, newer sensors are being developed for detection from saliva, sweat and interstitial fluid. These non-invasive sample matrices are more conducive to the development of wearable technologies for continuous physiological monitoring. In a recent example, a lithium-manganese oxide-modified electrode was used to detect lithium in artificial saliva.^53^ The sensor had an LOD of 50 *μ*M with a linear range up to 5 mM.

These sensors provide important foundations to selectively detect psychiatric drugs in complex matrices, but significant work remains to translate them to in-vivo, closed-loop monitoring in humans. Though a number of sporadic studies have been done to achieve in-vitro detection of psychiatric medications, more research is needed to develop more sensors for the simultaneous detection of all known psychiatric medications. Furthermore, these sensors need to be integrated into wearable device for continuous, physiological monitoring.

## Wearables for Mental Health Monitoring

In order to achieve continuous physiological monitoring of psychiatric medications, a critical component of a closed-loop feedback system that administers the proper amount of psychiatrics, sensors for psychiatric medications must be integrated into a wearable device. Electrochemical wearable sensors are well-established and have been used to detect glucose, lactate and electrolytes.^54^ The technology underlying these wearables is anticipated to be adaptable for drug monitoring. One study aimed at incorporating a non-invasive wearable device was for the detection of lithium in patients with bipolar disorder.^55^ A paper fluidics device with a stable reference electrode was used for detection from sweat. This platform utilizes a flexible electrochemical sensor comprised of a nanostructured, solid-state potentiometric sensor and a solid-contact reference electrode (RE) made of silver. The use of a polyvinyl chloride (PVC) membrane doped with an Ionic Liquid (IL) ensured the stability of the RE, showing “quasi-Nerstian behavior (56.8 +/− 3.9 mV/decade) in the range of clinical interest both in aqueous solution and in artificial sweat.” The paper fluidics aspect of the device consists of fast and slow adsorbing filter paper for sweat collection and disposal.

Further, in order for widespread clinical implementation of the technologies described here to occur, more comparisons between current methods and new technologies are necessary to ensure the validity of electrochemical signaling. Importantly, work to compare these has already begun. Using a reusable graphene-chitosan-coated electrode to analyze clozapine levels from clinical serum samples, Kang et al. demonstrated a fast and reliable electrochemical analysis that could aid in the dosing of schizophrenia medication.^56^ From their analysis of electrochemical and centralized laboratory methods, they found a high correlation to serum uric acid levels (r = 0.960, p < 0.001), demonstrating that electrochemical methods can provide a reliable point-of-care device for clinical applications.

Though electrochemical wearable therapeutic drug monitoring systems have been demonstrated to be viable options for clinical use, they must meet very high standards to ensure that they produce reliable results and provide optimal therapeutic outcomes.^57^ The stability of the devices is crucial, especially over long periods of time where drug monitoring may be needed, to prevent unwanted effects like biofouling and enzyme instability. Further, these devices must be highly selective for the chemical at-hand and validated.

Wearable, electrochemical monitoring of psychiatric medications poses an important future direction for the clinical space, especially due to the noninvasive and on-site screening capabilities these devices often have. As these electrochemical wearables become more prevalent, more and more future research and industry endeavors are likely to begin, providing patients and clinicians with new opportunities for innovative therapies.

## Conclusions

While there have been considerable advancements in in-vivo NT detection in animals, more work is needed to achieve equivalent detection in humans. This will require the development of nanosized electrodes that will elicit a minimal inflammatory response in the brain with extended stability to enable continuous measurements. Similarly, more work is needed to achieve in-vivo detection of psychiatric medications by integrating sensors into wearable devices. Once in-vivo detection of NT and psychiatric medications is achieved in humans, these detection modalities can be integrated into a closed-loop feedback system that administers the proper amount of psychiatric medication based on NT and medication levels. These sensors are critical to improve the diagnosis and treatment of mental disorders. Mental illnesses currently lack quantitative biomarkers that inform diagnosis and treatment. Verbal reporting of symptoms does not capture the inter-individua variability in biological mechanisms causing the symptoms. As a result, diagnosis and treatment of mental illness is highly subjective and error-prone, resulting in 30% of patients having treatment-resistant depression, for example.^58^

## Figures and Tables

**Figure 1.**
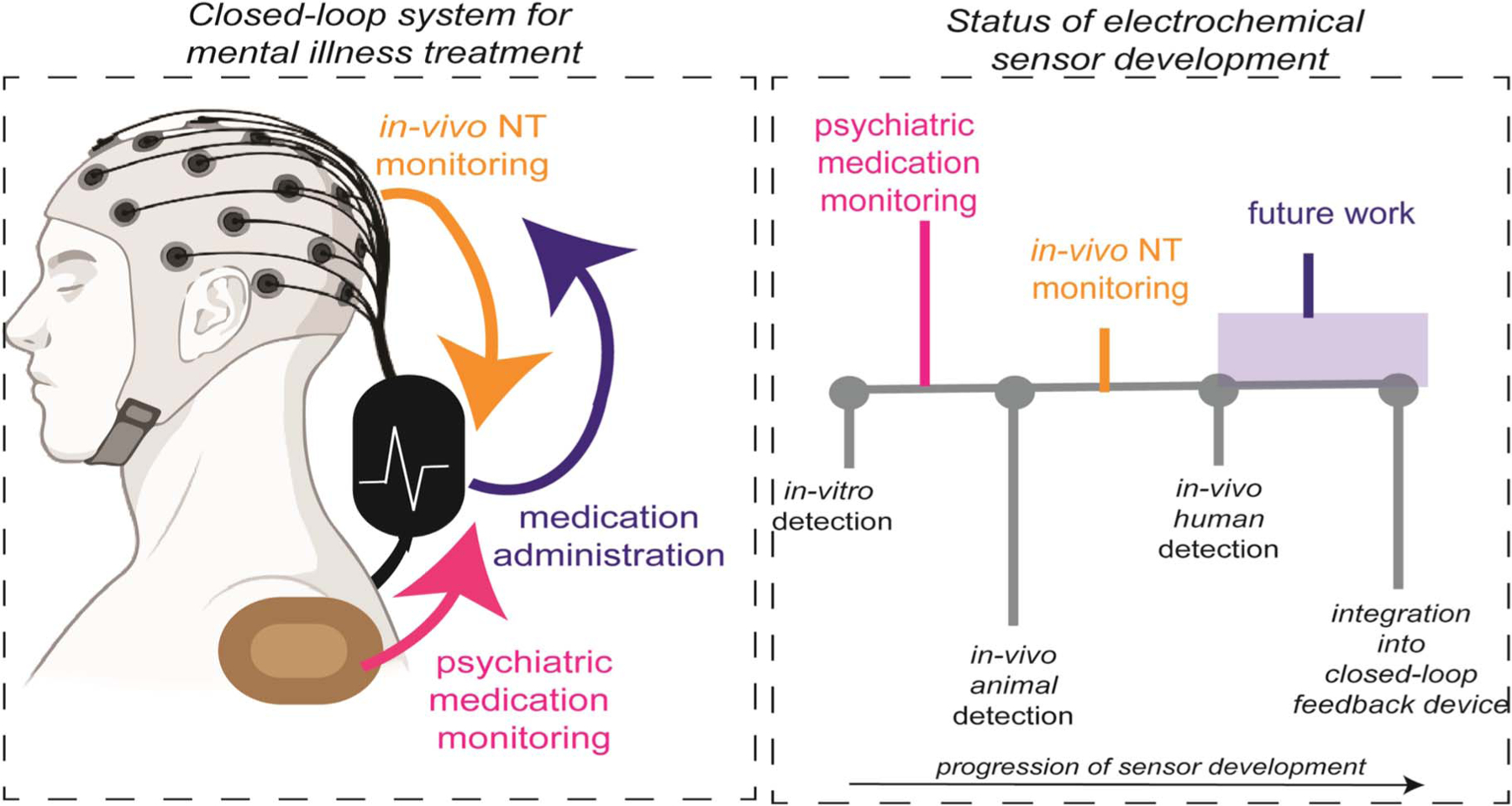
Electrochemical monitoring of NT and psychiatric medications as part of a closed-loop feedback system that administers psychiatric medication.

**Table I.** Electrochemical sensors for neurotransmitter detection.

Biosensor	NT	Sample	LOD	Linear Range	References
Direct	Dopamine	*In-vivo* mouse brain	50 nM	0.05 *μ*M–50 *μ*M	13
Direct	Serotonin	*In-vivo* mouse brain	20 nM	0.1 *μ*M–100 *μ*M	13
Enzymatic	Glutamate	*In-vivo* rat brain	44 nM	5 *μ*M–150 *μ*M	19
Enzymatic	GABA	*In-vivo* rodent brain	—	0–500 *μ*M	20
Enzymatic	Norepinephrine	Synthetic urine	196 nM	1–200 *μ*M	21
Enzymatic	Dopamine	*In-vivo* live rats	1 nM	10 nM–220 *μ*M	22
Enzymatic	Dopamine	Synthetic urine	670 nM	1.33–13.32 *μ*M	23
Enzymatic	Serotonin	*In-vitro*	48 nM	0.1–200 *μ*M	24
Aptameric	Dopamine	*In-vitro*	—	100 nM–5 *μ*M	27
Aptameric	Dopamine	*In-vitro*	60 pM	0.1 nM–10 nM	28
Aptameric	Glutamate	*In-*vitro artificial CSF	1.3 fM	10 fM–1 nM	29
Aptameric	Serotonin	*In-vitro* rat CSF	0.017 fM	1 pM–10 nM	30
